# Supplementary material for: A protein-based classifier for differentiating follicular thyroid adenoma and carcinoma
Source: EMBO Mol Med. 2025 May 29;17(7):1519–38. doi: 10.1038/s44321-025-00242-2 (PMC12254270; doi:10.1038/s44321-025-00242-2)
Supplement: Supplementary file 1 — Appendix [file 44321_2025_242_MOESM1_ESM.pdf]

Appendix

A protein-based classifier for differentiating follicular thyroid adenoma and carcinoma

Yaoting Sun, *et al*.

Table of contents

Appendix Figures

Appendix Figure S1. Discovery proteomics design and quality control. —Page 2

Appendix Figure S2. Comparison of machine learning models and feature counts performances. —Page 3

Appendix Figure S3. Protein feature characteristics. —Page 4 to Page 9

Appendix Tables

Appendix Table S1. Gene mutation frequency in FTA and FTC. —Page 10

Appendix Table S2. Twenty-four proteins established associations with thyroid physiology or pathology. —Page 11

Appendix Table S3. List of 66-gene panel of the thyroid cancer. —Page 12

Non-contributing We-TEC Investigators. —Page 13

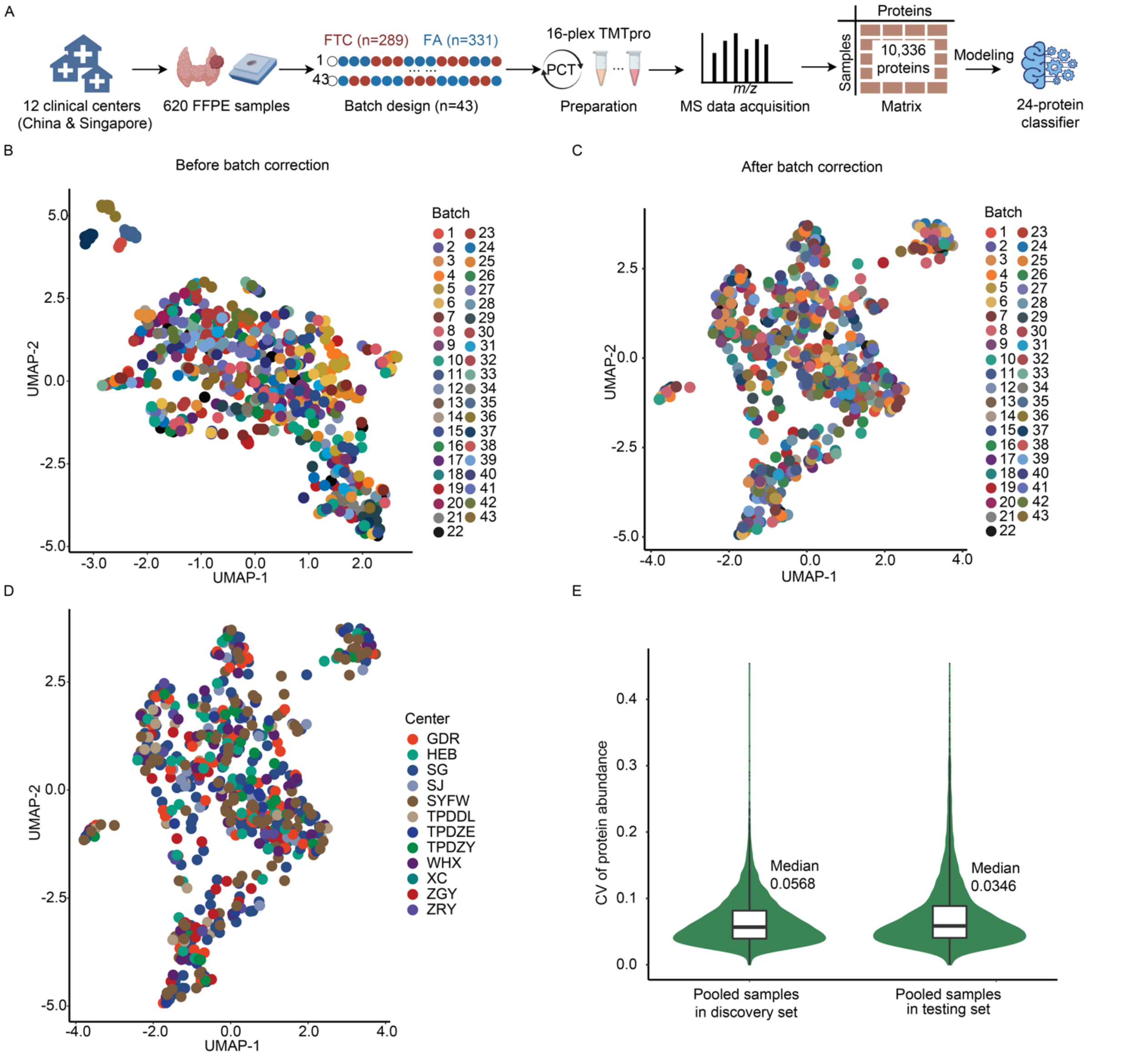

Appendix Figure S1. Discovery proteomics design and quality control.

Study flowchart. Samples (n=620) were retrospectively collected from 12 clinical centers. The samples were randomly allocated into 43 batches and prepared by pressure cycling technology (PCT) assisted sample preparation. Peptides were further labeled by 16-plex tandem mass tag (TMT) and data were acquired through the mass spectrometer with data-dependent acquisition (DDA) mode. There were 10,336 proteins quantified, and a 24-protein classifier was built based on them. (B-D) Uniform manifold approximation and projection (UMAP) plot showing the distribution of batches of samples using 7876 proteins (B) before and (C) after batch correction. Dots are colored according to the TMT batches. (D) UMAP analysis of clinical centers using 7876 proteins. (E) Violin plots show the CV of protein abundance across pooled samples in the discovery and testing sets. Medians are labeled upon the plot. The boxes represent the first and third quartiles, with the center line indicating the median. The whiskers extend to ±1.5 times the interquartile range, while the violin shapes depict the density of the data points.

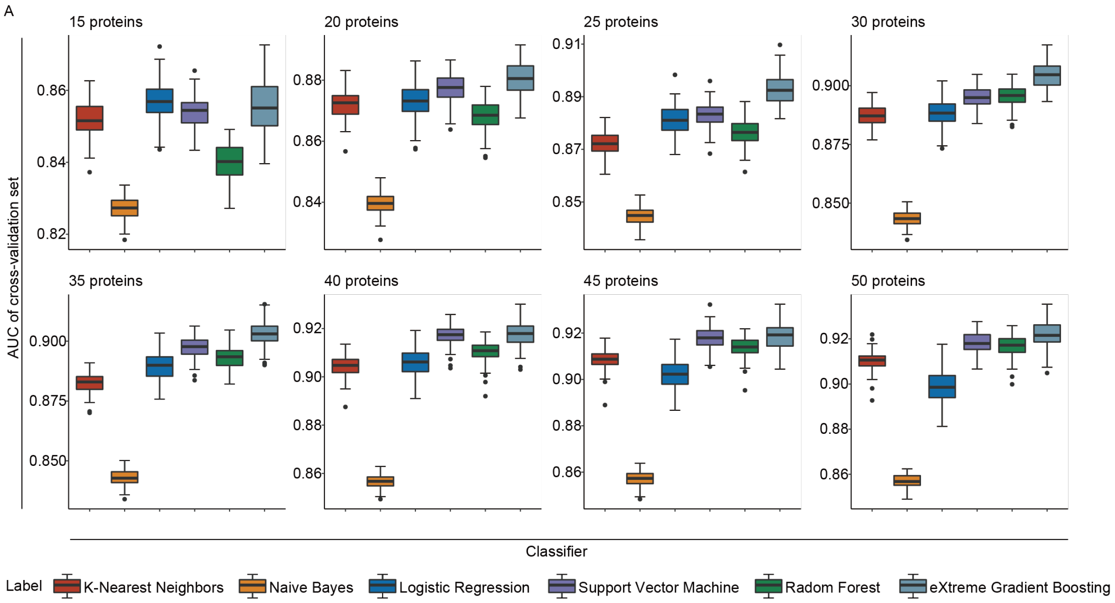

Appendix Figure S2. Comparison of machine learning models and feature counts performances.

(A)The compared six models (x-axis) are k-nearest neighbors (KNN), naive bayes (NB), linear regression (LR), support vector machine (SVM), random forest (RF), extreme gradient boosting (XGBoost). Each panel indicates the different protein feature counts applied**.** Each boxplot was derived from 100 times cross-validation on the training set. Y-axis showing the values of area under the curve (AUC) from the 100 iterations. The boxes represent the first and third quartiles, the center line indicates the median, the whiskers extend to ±1.5 times the interquartile range, and the dots represent individual data points.

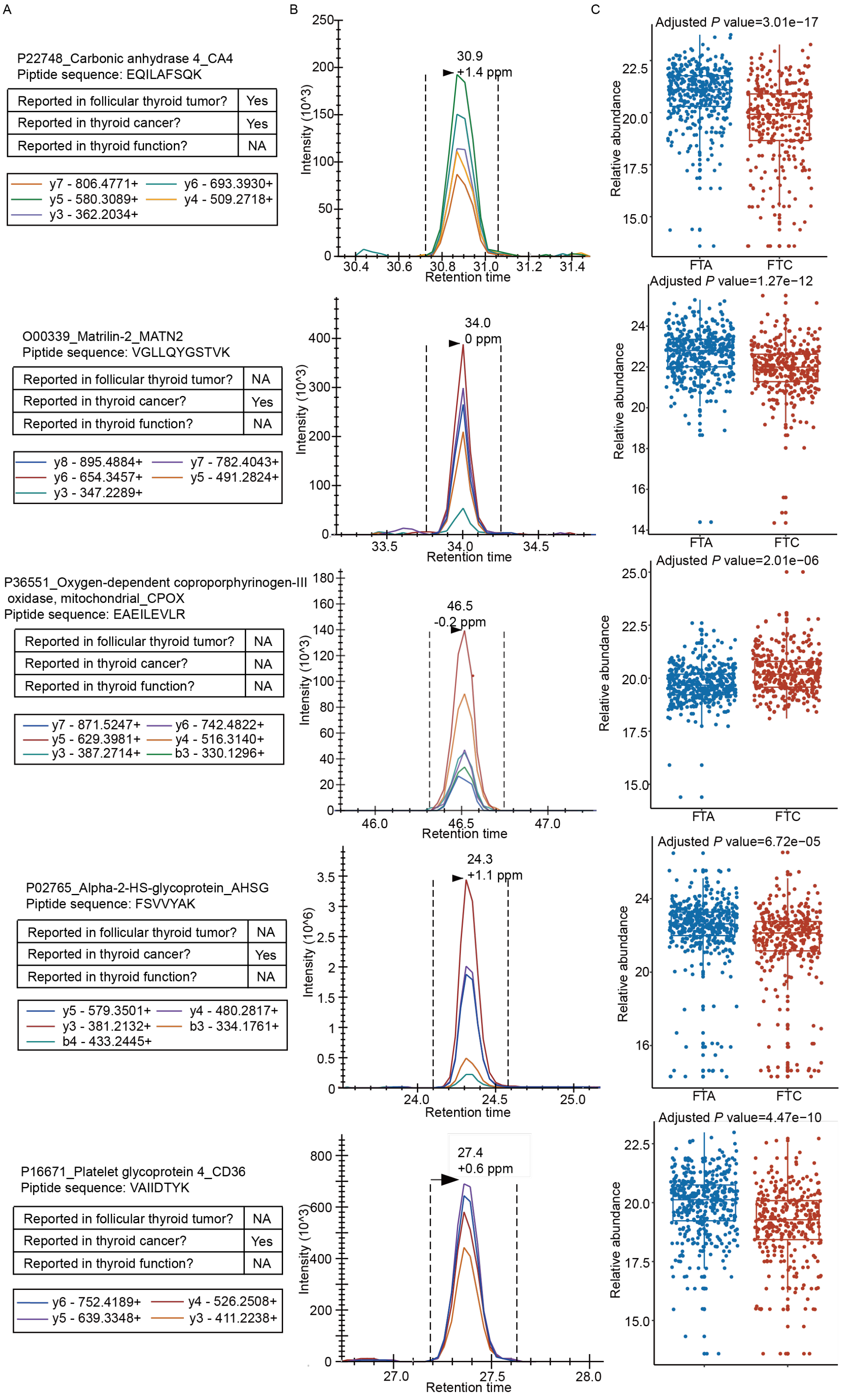
A
B
C
P22748_Carbonic anhydrase 4_CA4
Piptide sequence: EQILAFSQK
Reported in follicular thyroid tumor? Yes
Reported in thyroid cancer? Yes
Reported in thyroid function? NA
y7 - 806.4771+
y6 - 693.3930+
y5 - 580.3089+
y4 - 509.2718+
y3 - 362.2034+
30.9
+1.4 ppm
Intensity (10^3)
Retention time
Adjusted P value=3.01e−17
Relative abundance
FTA
FTC
O00339_Matrilin-2_MATN2
Piptide sequence: VGLLQYGSTVK
Reported in follicular thyroid tumor? NA
Reported in thyroid cancer? Yes
Reported in thyroid function? NA
y8 - 895.4884+
y7 - 782.4043+
y6 - 654.3457+
y5 - 491.2824+
y3 - 347.2289+
34.0
0 ppm
Adjusted P value=1.27e−12
P36551_Oxygen-dependent coproporphyrinogen-III oxidase, mitochondrial_CPOX
Piptide sequence: EAEILEVLR
Reported in follicular thyroid tumor? NA
Reported in thyroid cancer? NA
Reported in thyroid function? NA
y7 - 871.5247+
y6 - 742.4822+
y5 - 629.3981+
y4 - 516.3140+
y3 - 387.2714+
b3 - 330.1296+
46.5
-0.2 ppm
Adjusted P value=2.01e−06
P02765_Alpha-2-HS-glycoprotein_AHSG
Piptide sequence: FSVVYAK
Reported in follicular thyroid tumor? NA
Reported in thyroid cancer? Yes
Reported in thyroid function? NA
y5 - 579.3501+
y4 - 480.2817+
y3 - 381.2132+
b3 - 334.1761+
b4 - 433.2445+
24.3
+1.1 ppm
Intensity (10^6)
Adjusted P value=6.72e−05
P16671_Platelet glycoprotein 4_CD36
Piptide sequence: VAIIDTYK
Reported in follicular thyroid tumor? NA
Reported in thyroid cancer? Yes
Reported in thyroid function? NA
y6 - 752.4189+
y4 - 526.2508+
y5 - 639.3348+
y3 - 411.2238+
27.4
+0.6 ppm
Adjusted P value=4.47e−10

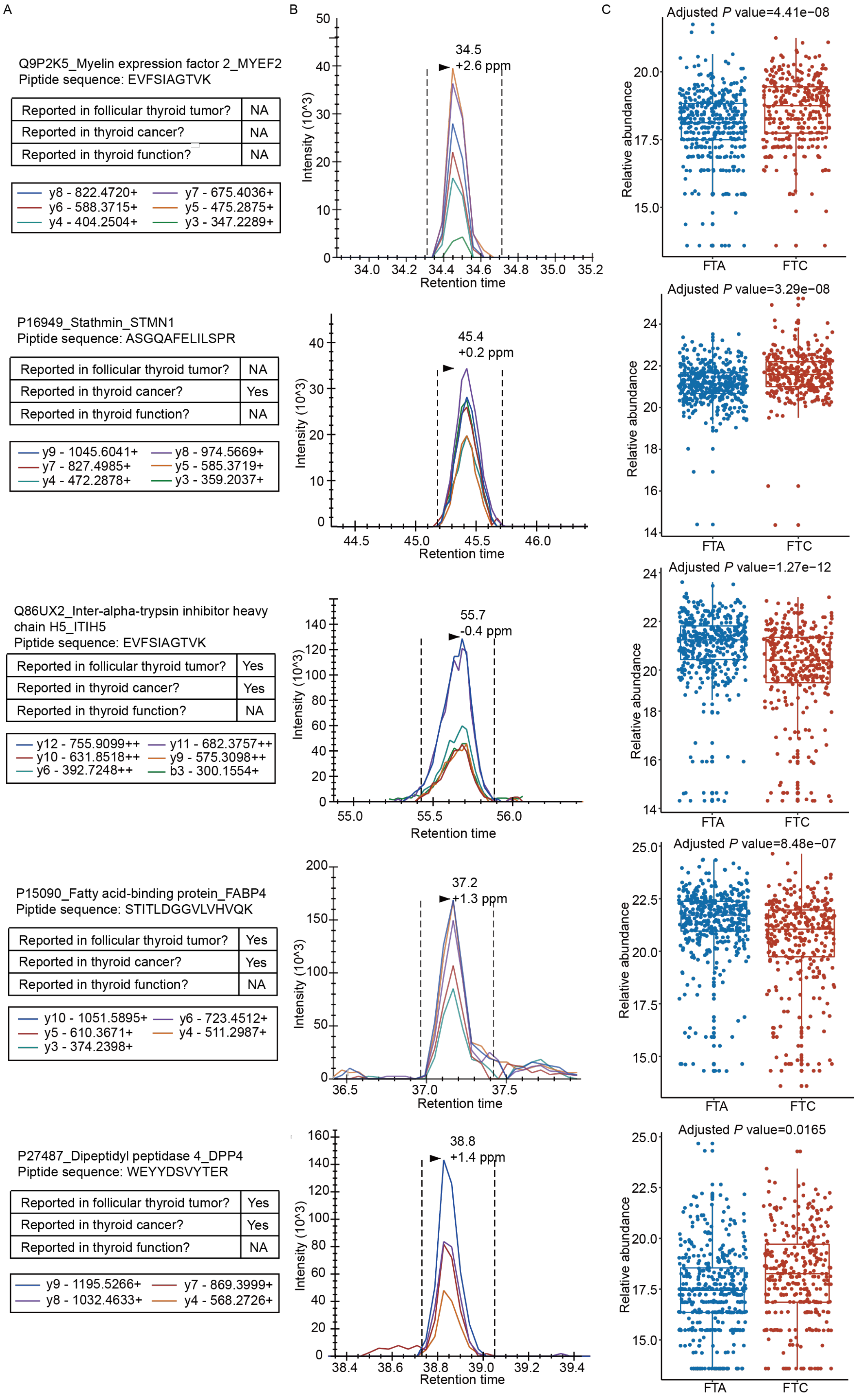

A
B
C
Q9P2K5_Myelin expression factor 2_MYEF2
Piptide sequence: EVFSIAGTVK
Reported in follicular thyroid tumor? NA
Reported in thyroid cancer? NA
Reported in thyroid function? NA
y8 - 822.4720+
y7 - 675.4036+
y6 - 588.3715+
y5 - 475.2875+
y4 - 404.2504+
y3 - 347.2289+
34.5
+2.6 ppm
Intensity (10^3)
Retention time
Adjusted P value=4.41e−08
Relative abundance
FTA
FTC
P16949_Stathmin_STMN1
Piptide sequence: ASGQAFELILSPR
Reported in follicular thyroid tumor? NA
Reported in thyroid cancer? Yes
Reported in thyroid function? NA
y9 - 1045.6041+
y8 - 974.5669+
y7 - 827.4985+
y5 - 585.3719+
y4 - 472.2878+
y3 - 359.2037+
45.4
+0.2 ppm
Adjusted P value=3.29e−08
Q86UX2_Inter-alpha-trypsin inhibitor heavy chain H5_ITIH5
Piptide sequence: EVFSIAGTVK
Reported in follicular thyroid tumor? Yes
Reported in thyroid cancer? Yes
Reported in thyroid function? NA
y12 - 755.9099++
y11 - 682.3757++
y10 - 631.8518++
y9 - 575.3098++
y6 - 392.7248++
b3 - 300.1554+
55.7
-0.4 ppm
Adjusted P value=1.27e−12
P15090_Fatty acid-binding protein_FABP4
Piptide sequence: STITLDGGVLVHVQK
Reported in follicular thyroid tumor? Yes
Reported in thyroid cancer? Yes
Reported in thyroid function? NA
y10 - 1051.5895+
y6 - 723.4512+
y5 - 610.3671+
y4 - 511.2987+
y3 - 374.2398+
37.2
+1.3 ppm
Adjusted P value=8.48e−07
P27487_Dipeptidyl peptidase 4_DPP4
Piptide sequence: WEYYDSVYTER
Reported in follicular thyroid tumor? Yes
Reported in thyroid cancer? Yes
Reported in thyroid function? NA
y9 - 1195.5266+
y7 - 869.3999+
y8 - 1032.4633+
y4 - 568.2726+
38.8
+1.4 ppm
Adjusted P value=0.0165

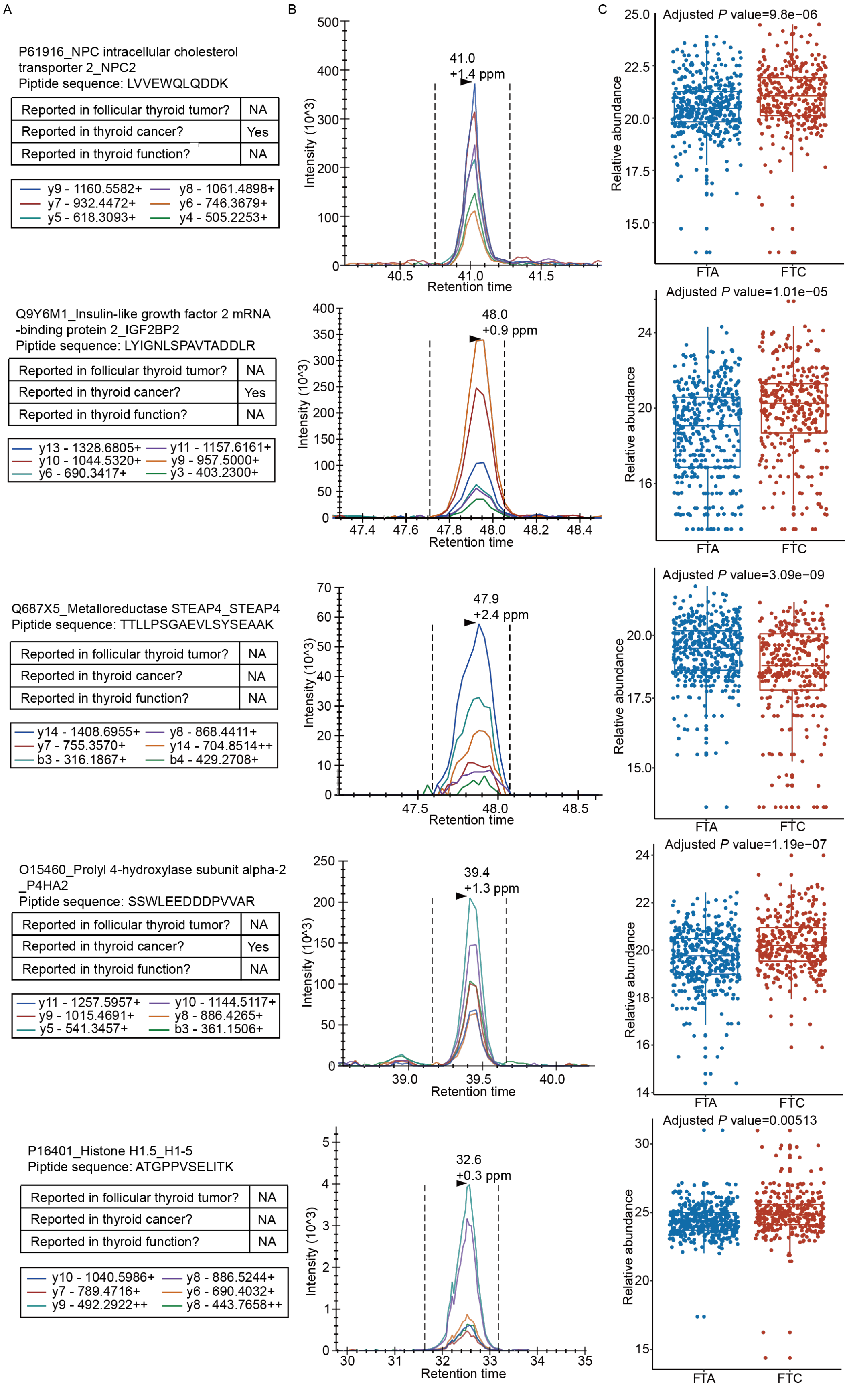
A
B
C
P61916_NPC intracellular cholesterol transporter 2_NPC2
Piptide sequence: LVVEWQLQDDK
Reported in follicular thyroid tumor? NA
Reported in thyroid cancer? Yes
Reported in thyroid function? NA
y9 - 1160.5582+
y8 - 1061.4898+
y7 - 932.4472+
y6 - 746.3679+
y5 - 618.3093+
y4 - 505.2253+
41.0
+1.4 ppm
Intensity (10^3)
Retention time
Adjusted P value=9.8e−06
Relative abundance
FTA
FTC
Q9Y6M1_Insulin-like growth factor 2 mRNA -binding protein 2_IGF2BP2
Piptide sequence: LYIGNLSPAVTADDLR
Reported in follicular thyroid tumor? NA
Reported in thyroid cancer? Yes
Reported in thyroid function? NA
y13 - 1328.6805+
y11 - 1157.6161+
y10 - 1044.5320+
y9 - 957.5000+
y6 - 690.3417+
y3 - 403.2300+
48.0
+0.9 ppm
Adjusted P value=1.01e−05
Q687X5_Metalloreductase STEAP4_STEAP4
Piptide sequence: TTLLPSGAEVLSYSEAAK
Reported in follicular thyroid tumor? NA
Reported in thyroid cancer? NA
Reported in thyroid function? NA
y14 - 1408.6955+
y8 - 868.4411+
y7 - 755.3570+
y14 - 704.8514++
b3 - 316.1867+
b4 - 429.2708+
47.9
+2.4 ppm
Adjusted P value=3.09e−09
O15460_Prolyl 4-hydroxylase subunit alpha-2 _P4HA2
Piptide sequence: SSWLEEDDDPVVAR
Reported in follicular thyroid tumor? NA
Reported in thyroid cancer? Yes
Reported in thyroid function? NA
y11 - 1257.5957+
y10 - 1144.5117+
y9 - 1015.4691+
y8 - 886.4265+
y5 - 541.3457+
b3 - 361.1506+
39.4
+1.3 ppm
Adjusted P value=1.19e−07
P16401_Histone H1.5_H1-5
Piptide sequence: ATGPPVSELITK
Reported in follicular thyroid tumor? NA
Reported in thyroid cancer? NA
Reported in thyroid function? NA
y10 - 1040.5986+
y8 - 886.5244+
y7 - 789.4716+
y6 - 690.4032+
y9 - 492.2922++
y8 - 443.7658++
32.6
+0.3 ppm
Adjusted P value=0.00513

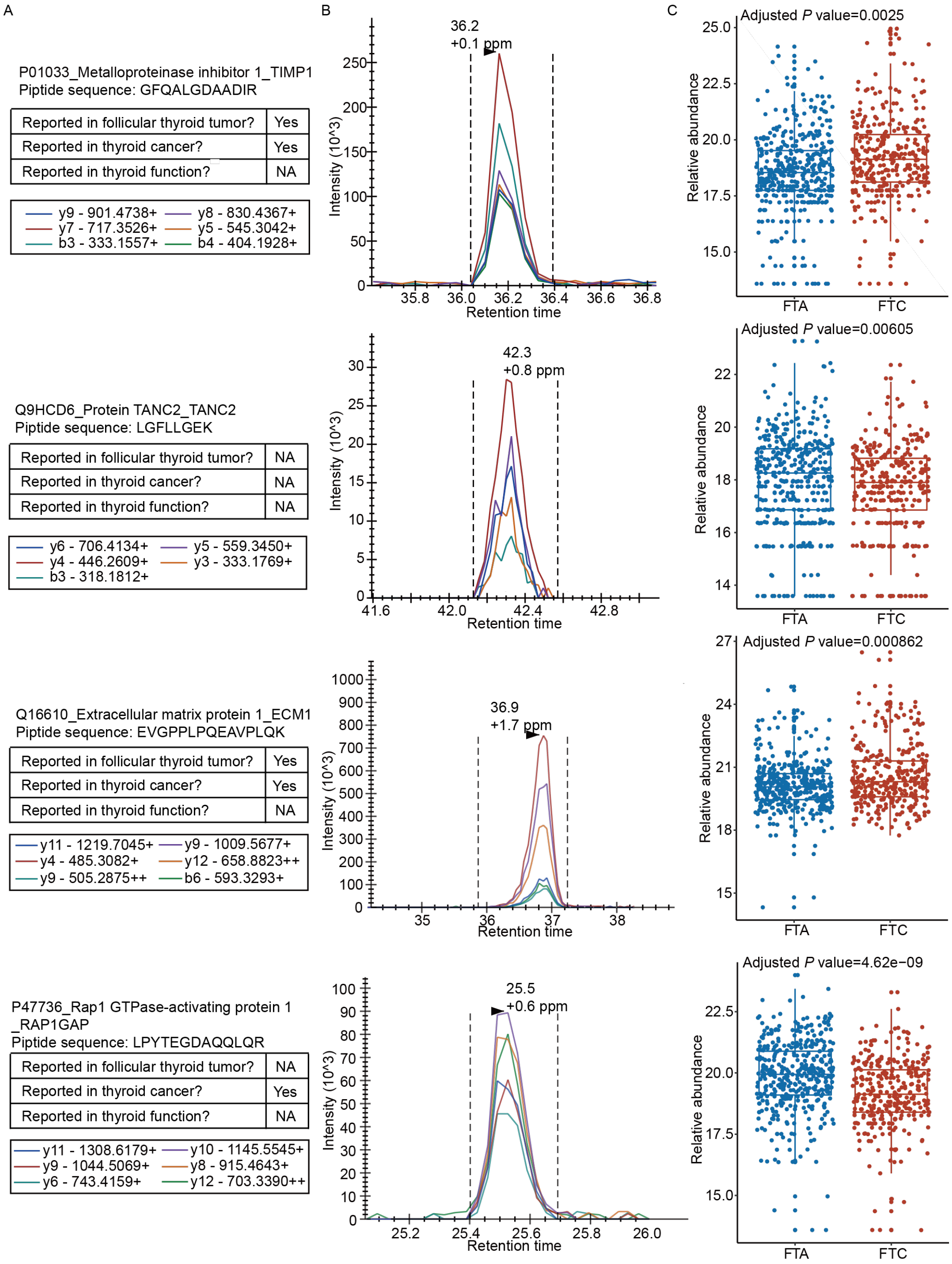
A
B
C
P01033_Metalloproteinase inhibitor 1_TIMP1
Piptide sequence: GFQALGDAADIR
Reported in follicular thyroid tumor? Yes
Reported in thyroid cancer? Yes
Reported in thyroid function? NA
y9 - 901.4738+
y8 - 830.4367+
y7 - 717.3526+
y5 - 545.3042+
b3 - 333.1557+
b4 - 404.1928+
36.2
+0.1 ppm
Intensity (10^3)
Retention time
Adjusted P value=0.0025
Relative abundance
FTA
FTC
Q9HCD6_Protein TANC2_TANC2
Piptide sequence: LGFLLGEK
Reported in follicular thyroid tumor? NA
Reported in thyroid cancer? NA
Reported in thyroid function? NA
y6 - 706.4134+
y5 - 559.3450+
y4 - 446.2609+
y3 - 333.1769+
b3 - 318.1812+
42.3
+0.8 ppm
Intensity (10^3)
Retention time
Adjusted P value=0.00605
Relative abundance
FTA
FTC
Q16610_Extracellular matrix protein 1_ECM1
Piptide sequence: EVGPPLPQEAVPLQK
Reported in follicular thyroid tumor? Yes
Reported in thyroid cancer? Yes
Reported in thyroid function? NA
y11 - 1219.7045+
y9 - 1009.5677+
y4 - 485.3082+
y12 - 658.8823++
y9 - 505.2875++
b6 - 593.3293+
36.9
+1.7 ppm
Intensity (10^3)
Retention time
Adjusted P value=0.000862
Relative abundance
FTA
FTC
P47736_Rap1 GTPase-activating protein 1
_RAP1GAP
Piptide sequence: LPYTEGDAQQLQR
Reported in follicular thyroid tumor? NA
Reported in thyroid cancer? Yes
Reported in thyroid function? NA
y11 - 1308.6179+
y10 - 1145.5545+
y9 - 1044.5069+
y8 - 915.4643+
y6 - 743.4159+
y12 - 703.3390++
25.5
+0.6 ppm
Intensity (10^3)
Retention time
Adjusted P value=4.62e−09
Relative abundance
FTA
FTC

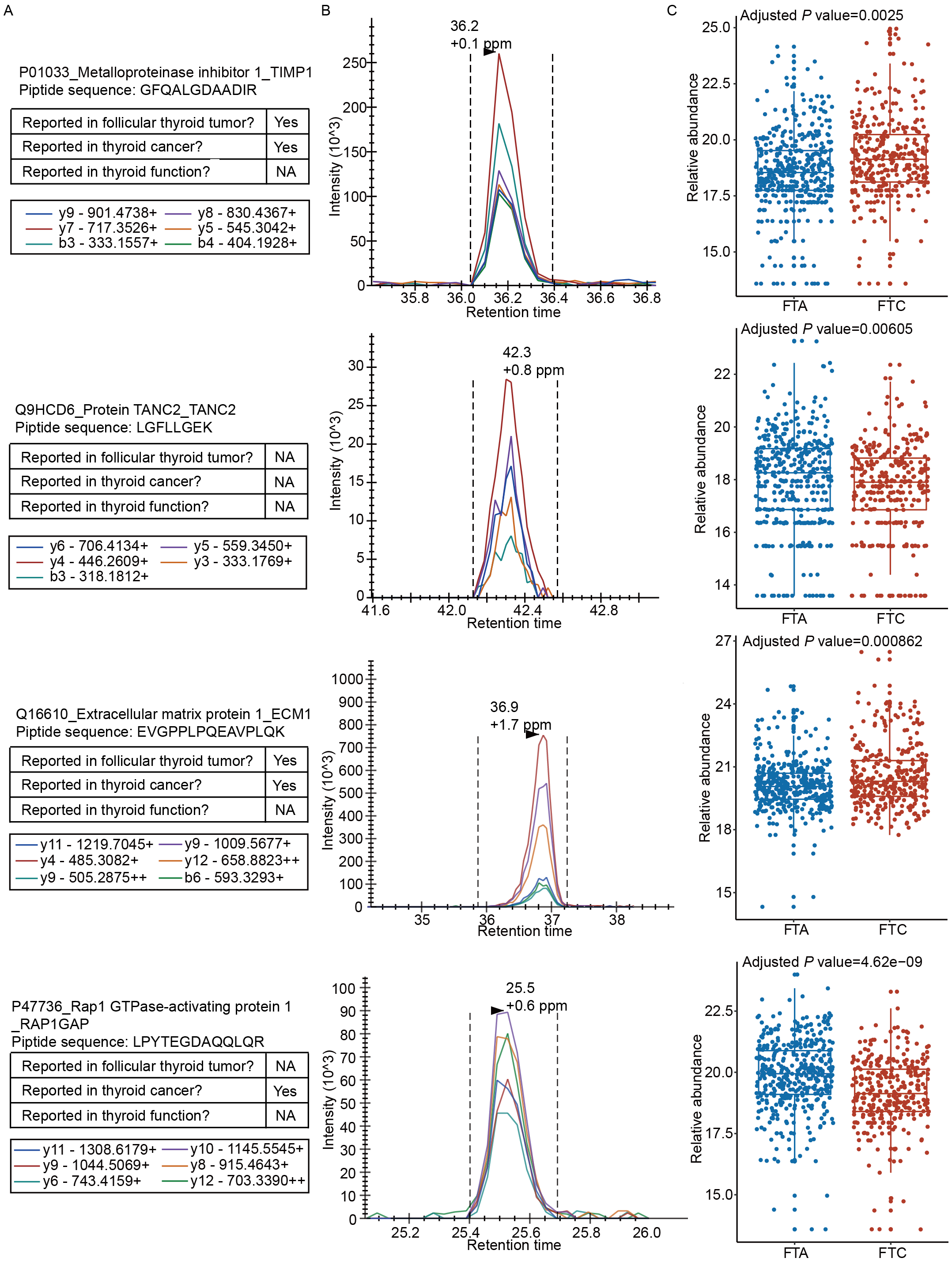

Appendix Figure S3. Protein feature characteristics.

(A) The protein identified by Uniprot ID_protein name_gene name with corresponding sequences which detected by targeted proteomics. (B) A chromatographic profile of a representative peptide precursor peak group. (C) Boxplots showing the protein abundance of each sample measured by parallel-reaction monitoring. FTA (n=415) and FTC (n=314). The boxes represent the first and third quartiles, the center

line indicates the median, the whiskers extend to ±1.5 times the interquartile range, and the dots represent individual data points. *P* values are calculated by two-sided Welch's *t*-test.

Appendix Table S1. Gene mutation frequency in FTA and FTC.

Gene name	FTA	FTC
NRAS	12.4%	21.6%
HRAS	7.3%	14.1%
TERT	2.3%	18.8%
DICER1	4.0%	10.3%
EIF1AX	5.8%	6.6%
KRAS	2.8%	3.8%
TSHR	3.0%	2.8%
TP53	0.8%	5.6%
BRAF	2.5%	2.8%
SPOP	2.5%	1.9%
EZH1	2.0%	0.0%
CTNNB1	0.5%	1.9%
GLIS3	1.0%	1.9%
RET	0.5%	1.9%
MEN1	0.5%	1.9%
GNAS	0.5%	0.5%
KMT2C	0.3%	1.4%
NOTCH1	0.3%	1.4%
PIK3CA	0.8%	0.9%
POR	1.0%	0.5%
KMT2D	0.8%	0.5%
PTEN	0.3%	0.9%
ALK	0.8%	0.0%
NTRK1	0.8%	0.0%
RB1	0.3%	0.9%
STK11	0.3%	0.9%
APC	0.3%	0.5%
CDKN2A	0.3%	0.0%
LRP1B	0.3%	0.5%
VHL	0.0%	0.9%
AKT1	0.0%	0.5%
CDK12	0.0%	0.5%
EGFR	0.3%	0.0%
FAM193A	0.3%	0.0%
FGFR4	0.0%	0.5%
IDH1	0.3%	0.0%
KIT	0.3%	0.0%
MET	0.3%	0.0%
NCOR2	0.0%	0.5%
ROS1	0.3%	0.0%
TSC2	0.3%	0.0%

Appendix Table S2. Twenty-four proteins established associations with thyroid physiology or pathology.

Rank	Uniprot ID	Protein name	Gene name	Follicular tumour related	Thyroid cancer related	Thyroid function related
1	P22748	Carbonic anhydrase 4	*CA4*	Yes	-	-
2	O00339	Matrilin-2	*MATN2*	-	Yes	-
3	P36551	Oxygen-dependent coproporphyrinogen-III oxidase	*CPOX*	-	-	-
4	P02765	Alpha-2-HS-glycoprotein	*AHSG*	-	Yes	-
5	P16671	Platelet glycoprotein 4	*CD36*	-	Yes	-
6	Q9P2K5	Myelin expression factor 2	*MYEF2*	-	-	-
7	P16949	Stathmin	*STMN1*	-	Yes	-
8	Q86UX2	Inter-alpha-trypsin inhibitor heavy chain H5	*ITIH5*	Yes	Yes	-
9	P15090	Fatty acid-binding protein	*FABP4*	Yes	Yes	Yes
10	P27487	Dipeptidyl peptidase 4	*DPP4*	Yes	Yes	-
11	P61916	NPC intracellular cholesterol transporter 2	*NPC2*	-	Yes	-
12	Q9Y6M1	Insulin-like growth factor 2 mRNA-binding protein 2	*IGF2BP2*	-	Yes	-
13	Q687X5	Metalloreductase STEAP4	*STEAP4*	-	-	-
14	O15460	Prolyl 4-hydroxylase subunit alpha-2	*P4HA2*	-	Yes	-
15	P16401	Histone H1.5	*H1-5*	-	-	-
16	Q86XX4	Extracellular matrix organizing protein FRAS1	*FRAS1*	-	-	-
17	P98164	Low-density lipoprotein receptor-related protein 2	*LRP2*	-	Yes	Yes
18	Q8N6C5	Immunoglobulin superfamily member 1	*IGSF1*	-	Yes	Yes
19	P29762	Cellular retinoic acid-binding protein 1	*CRABP1*	Yes	Yes	-
20	P52926	High mobility group protein HMGI-C	*HMGA2*	Yes	Yes	-
21	P01033	Metalloproteinase inhibitor 1	*TIMP1*	Yes	Yes	-
22	Q9HCD6	Protein TANC2	*TANC2*	-	-	-
23	Q16610	Extracellular matrix protein 1	*ECM1*	Yes	Yes	-
24	P47736	Rap1 GTPase-activating protein 1	*RAP1GAP*	-	Yes	-

Appendix Table S3. List of 66-gene panel of the thyroid cancer.

Gene List					
AKT1	*EGFR*	*FLT3*	*KRAS*	*NTRK3*	*SMAD4*
ALK	*EIF1AX*	*GLIS3*	*LRP1B*	*PIK3CA*	*SPOP*
APC	*EP300*	*GNAQ*	*MEN1*	*PLEKHS1*	*STK11*
ATM	*ERBB4*	*GNAS*	*MET*	*POR*	*SUGCT*
BANP	*EZH1*	*HRAS*	*MTOR*	*PPARG*	*TERT*
BRAF	*FAM193A*	*IDH1*	*NCOR2*	*PTEN*	*TP53*
CDK12	*FARSB*	*IDH2*	*NF1*	*PTH*	*TRIM61*
CDKN2A	*FGFR1*	*KIT*	*NF2*	*RB1*	*TSC2*
CHEK2	*FGFR2*	*KLK1*	*NOTCH1*	*RBM10*	*TSHR*
CTNNB1	*FGFR3*	*KMT2C*	*NRAS*	*RET*	*VHL*
DICER1	*FGFR4*	*KMT2D*	*NTRK1*	*ROS1*	*ZNF148*

Non-contributing We-TEC Investigators

Bo Huang[1], Gaosong Wu[2], Guan Ruan[3], Guang Chen[4], Guoyang Wu[5], Huixiong Xu[6], James A. Fagin[7], Jianhua Wang[8], Jing Liu[9], Lei Liang[10], Meiping Shen[11], Qiushi Zhang[3], Wen Tian[12], Xianghui He[13], Xiao Shi[14], Yan Li[15]

[1]Liaoning Cancer Hospital & Institute, Cancer Hospital of Dalian University of Technology, Shenyang, China;

[2]Department of Thyroid and Breast Surgery, Zhongnan Hospital of Wuhan University, Wuhan, China;

[3]Westlake Omics (Hangzhou) Biotechnology Co., Ltd., Hangzhou, China.

[4]Department of Thyroid Surgery, General Surgery Center, The First Hospital of Jilin University, Changchun, China;

[5]Department of General Surgery, Zhongshan Hospital Affiliated with Xiamen University, Xiamen, China;

[6]Department of Ultrasound, Zhongshan Hospital, Institute of Ultrasound in Medicine and Engineering, Fudan University, Shanghai, China;

[7]Memorial Sloan Kettering Cancer Center, New York, USA;

[8]Department of Thyroid and Breast Surgery, Affiliated Hospital of Integrated Chinese and Western, Nanjing University of Chinese Medicine, Nanjing, China;

[9]First Hospital of Shanxi Medical University, Thyroid Surgery Department, Taiyuan, China;

[10]Department of Ultrasound, Aerospace Center Hospital, Beijing, China;

[11]Department of General Surgery, the First Affiliated of Nanjing Medical University, Nanjing, China;

[12]Department of Thyroid & Hernia Surgery, Medical Department of General Surgery, The First Medical Center of Chinese People's Liberation Army General Hospital, Beijing, China;

[13]Department of General Surgery, Tianjin Medical University General Hospital, Tianjin, China;

[14]Department of Head and Neck Surgery, Fudan University Shanghai Cancer Centre; Department of Oncology, Shanghai Medical College, Fudan University, Shanghai, China;

[15]College of Basic Medical Sciences, Shanghai Jiao Tong University, Shanghai, China;
