# Supplementary material for: A protein-based classifier for differentiating follicular thyroid adenoma and carcinoma
Source: EMBO Mol Med. 2025 May 29;17(7):1519–38. doi: 10.1038/s44321-025-00242-2 (PMC12254270; doi:10.1038/s44321-025-00242-2)
Supplement: Supplementary file 4 — Expanded View Figures [file 44321_2025_242_MOESM4_ESM.pdf]

Expanded View Figures

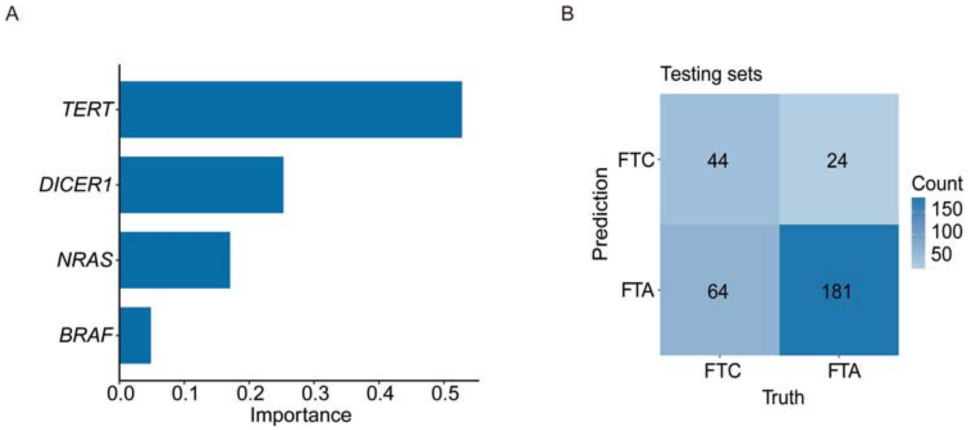

Figure EV1. Features and evaluation result of gene-based model.

(**A**) The importance rank of four selected features. (**B**) Confusion matrix of the four-gene model.

© The Author(s)

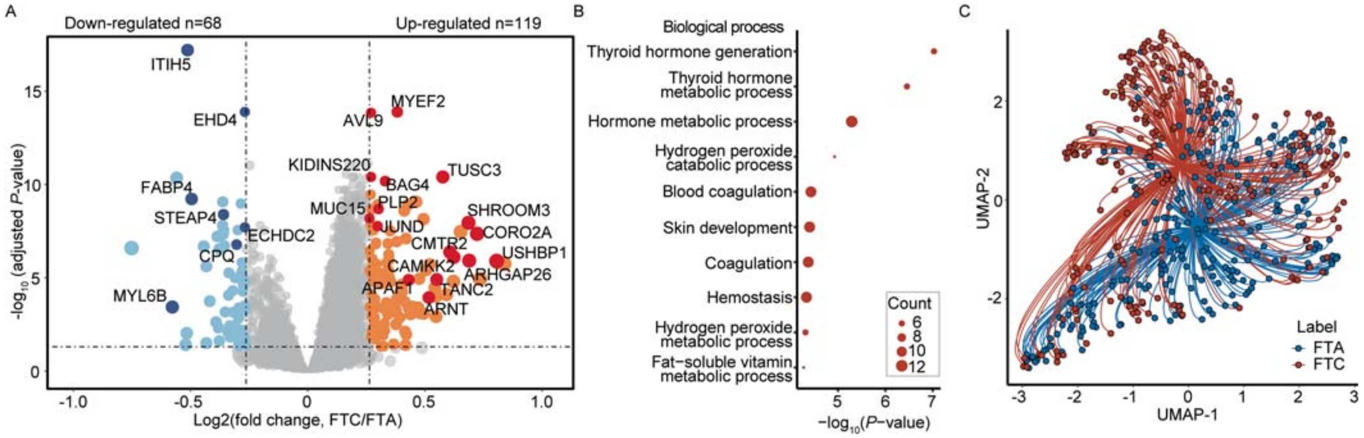

Figure EV2. Comparative proteomic analysis for FTC vs. FTA in TMT discovery dataset.

(**A**) Differentially expressed proteins (DEPs) in FTC ($n = 224$) and FTA ($n = 261$). Thresholds of significantly dysregulated proteins: fold change >1.2 with Benjamini & Hochberg adjusted $P < 0.05$ (Welch's *t*-test). The highlighted proteins are the features selected by the model in Fig. EV3. (**B**) Gene ontology (GO) biological process enrichment. X-axis represents the *P* and node size indicates the protein count of enriched items. *P* values are calculated using a one-sided Fisher's Exact Test. (**C**) UMAP plot showing FA and FTC are partially resolved using 187 DEPs.

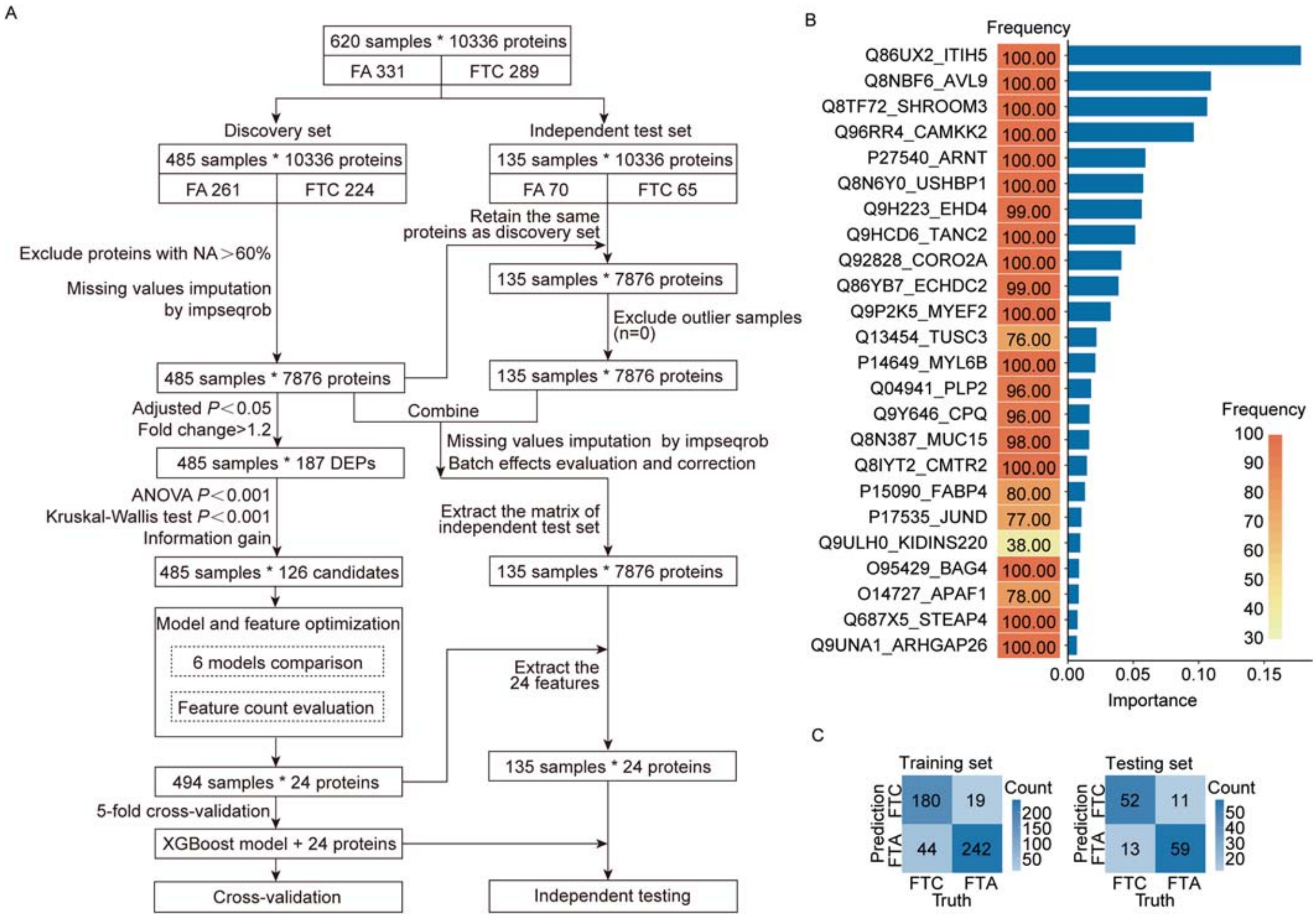

Figure EV3. Modeling using the discovery proteomic data.

(**A**) Schematic of XGBoost model construction. Samples firstly are divided into a discovery set and an independent set. Feature selection and model training are based on the discovery set and model performance evaluation is based on the independent test set. (**B**) Importance ranking of selected protein features and the frequency of 24 feature proteins when conducting 100 times feature selection on the training set. The orange color bar shows the selection frequency. (**C**) Confusion matrix of the 24-protein classifier. The blue color bar indicates the sample counts.

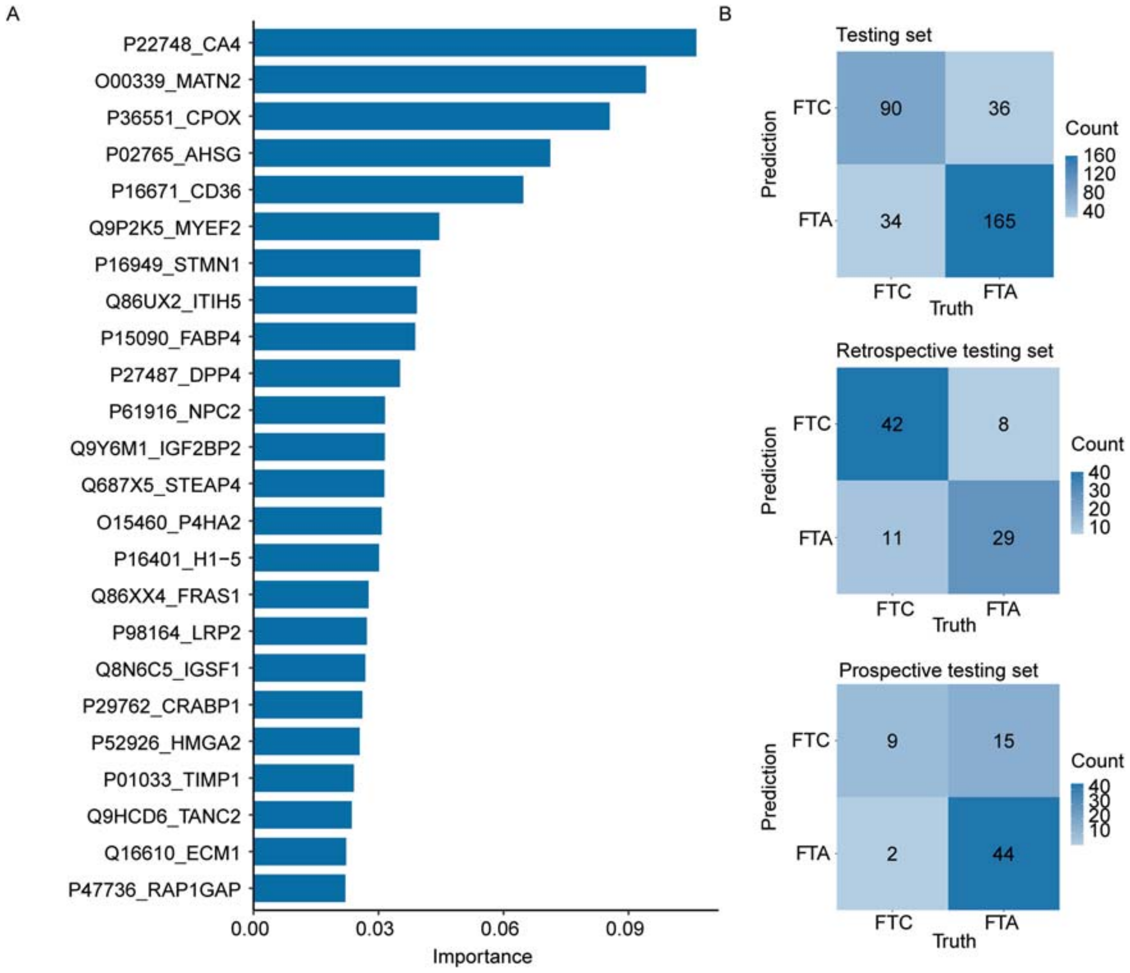

Figure EV4. Targeted proteomic data-based feature importance and model performance.

(**A**) The 24 selected targeted protein features and their importance rankings. (**B**) Confusion matrix of the 24-protein classifier. Colors indicate the sample counts.

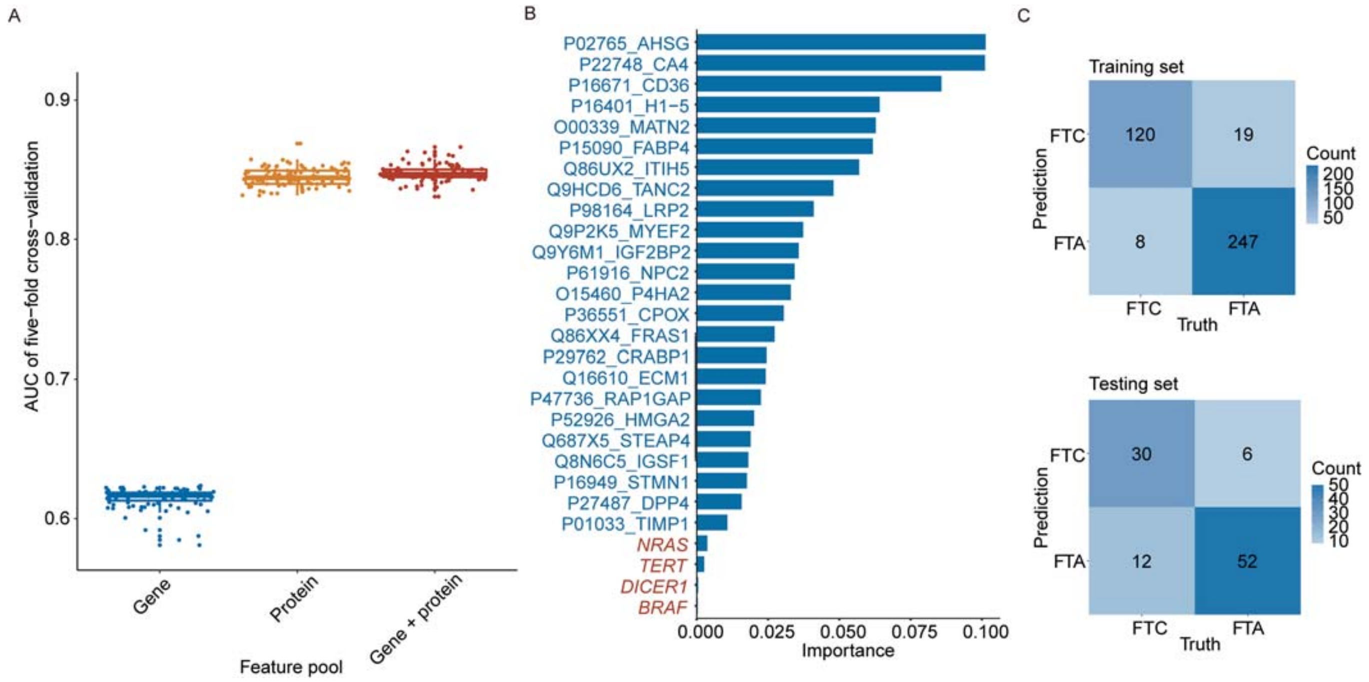

Figure EV5. Model performance comparison and characters of the combined feature-based model.

(**A**) The area under the curve (AUC) comparison of gene-, protein-, gene and protein-based models of five-fold cross-validation for one-hundred-time iterations. Each dot indicates one iteration. The box plots ($n = 100$ per group) are defined as follows: the lower and upper edges of the box represent the 25th percentile (first quartile) and the 75th percentile (third quartile), respectively; the central line represents the 50th percentile (median); and the whiskers span from the 0th percentile (minimum) to the 100th percentile (maximum). (**B**) The importance ranking of features. Protein features are colored in blue and gene features are colored in red. (**C**) Confusion matrix of the combined feature classifier. Colors indicate the sample counts.

© The Author(s)
